# Tracing Evolutionary Footprints to Identify Novel Gene Functional Linkages

**DOI:** 10.1371/journal.pone.0066817

**Published:** 2013-06-25

**Authors:** Yong Chen, Li Yang, Yunfeng Ding, Shuyan Zhang, Tong He, Fenglou Mao, Congyan Zhang, Huina Zhang, Chaoxing Huo, Pingsheng Liu

**Affiliations:** 1 National Laboratory of Biomacromolecules, Institute of Biophysics, Chinese Academy of Sciences, Beijing, China; 2 University of Chinese Academy of Sciences, Beijing, China; 3 School of Applied Mathematics, Central University of Finance and Economics, Beijing, China; 4 Computational Systems Biology Laboratory, Department of Biochemistry and Molecular Biology, Institute of Bioinformatics, University of Georgia, Athens, Georgia, United States of America; University of Lausanne, Switzerland

## Abstract

Systematic determination of gene function is an essential step in fully understanding the precise contribution of each gene for the proper execution of molecular functions in the cell. Gene functional linkage is defined as to describe the relationship of a group of genes with similar functions. With thousands of genomes sequenced, there arises a great opportunity to utilize gene evolutionary information to identify gene functional linkages. To this end, we established a computational method (called TRACE) to trace gene footprints through a gene functional network constructed from 341 prokaryotic genomes. TRACE performance was validated and successfully tested to predict enzyme functions as well as components of pathway. A so far undescribed chromosome partitioning-like protein ro03654 of an oleaginous bacteria *Rhodococcus sp.* RHA1 (RHA1) was predicted and verified experimentally with its deletion mutant showing growth inhibition compared to RHA1 wild type. In addition, four proteins were predicted to act as prokaryotic SNARE-like proteins, and two of them were shown to be localized at the plasma membrane. Thus, we believe that TRACE is an effective new method to infer prokaryotic gene functional linkages by tracing evolutionary events.

## Introduction

Systematic, genome-wide understanding of gene function is a fundamental and long-standing, yet so far unaccomplished goal in both pro- and eukaryotes. More than 2,000 prokaryotic genomes have been sequenced and collected in the NCBI databank (Release of July 2012) [1], but for many of these genomes, close to 40% of the genes functions are not characterized. A gene functional linkage is defined as a set of genes that are of similar function, or which are involved in the same biological process [2]–[4]. Whilst some gene function analysis has been performed using computational prediction methods as well as experimental characterization, lack of powerful analytical tools have so far prevented a more systematic search for gene functional correlations [5]–[7].

The explosion of sequenced genomes across the phyla provides a unique opportunity to investigate gene functional relationships by comparative genome analysis. Methods of comparative genome data mining have recently emerged for automatic identification of functional associations, and for assigning putative roles of yet unannotated genes in both pro- and eukaryotic organisms [8]–[10]. Among the currently available tools, the most commonly used method is based on mapping homologous sequences to genes of well-defined function. However, such homology-based prediction excludes analysis of genes with no sequence similarity [11], [12]. To overcome these limitations, methods were proposed previously to assign putative gene function based on the genomic context, including gene clusters [13]–[16], gene neighbours [17], [18], as well as phylogenetic profiling [6], [19], [20]. The phylogenetic profile method (PPM) is a widely accepted analysis tool that utilizes genomic context to identify gene functional linkages [3], [4], [6]. The underlying assumption of this method is that evolutionary co-occurrence of genes across organisms indicates a correlation in function. Clusters of genes with matching evolutionary profiles are subsequently subjected to pathway or biological module analysis [4], [21].

By utilizing multiple layers of biological information, these methods have greatly contributed to the recent progress made in uncovering of gene functional linkages. However, they are usually limited in terms of applications and technical calculations [6], [22]. A technical problem of PPM is that it transforms gene similarity into a 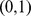 vector, and then calculates the correlation of phylogenetic profiles. This method of data processing omits special evolutionary events observed for individual genes, resulting in a high degree of information loss. Thus, an effective method for identification of putative function of unannotated genes and functional linkages is desirable that has both the ability of genome-wide prediction as well as utilizing evolutionary information for individual genes [6], [23].

To this aim, we have developed a novel computational method TRACE specifically for prokaryotic organisms. We used TRACE to detect functional linkages by tracing gene evolutionary events (at the operon and domain level) in 341 prokaryotic genomes. Initially, TRACE utilizes the genes of 341 genomes to construct a gene functional network. Subsequently, it calculates the value of the shortest path distance of a gene pair as their functional distance. All genes in the same genome can be prioritized by the functional distances to a given gene to find its linkages. Validation results show that TRACE was able to obtain very high precision in predicting specific enzyme functions and components of pathways. We predicted and experimentally verified novel functions and linkages of 5 hypothetical proteins of *Rhodococcus sp.* RHA1, an oleaginous bacterium, which has super ability of lipid synthesis and metabolism [24]–[26]. The gene ro03654 (GI 111020643) of RHA1 was successfully predicted to encode a chromosome partitioning-like protein. The function of the protein was verified using genetic and biochemical approaches. Compared with wild type, a ro03654 deletion mutant exhibited significantly lower growth rates. In addition, four genes, ro05535 (GI 111022501), ro05534 (GI 111022500), ro03137 (GI 111020126) and ro08552 (GI 111025334) were predicted to encode SNARE-like proteins. ro05535-EGFP and ro03137-EGFP fusion proteins were localized to the plasma membrane of RHA1 cells, showing SNARE protein characteristics.

## Results

### Analysis of TRACE performance

**Figure 1:**
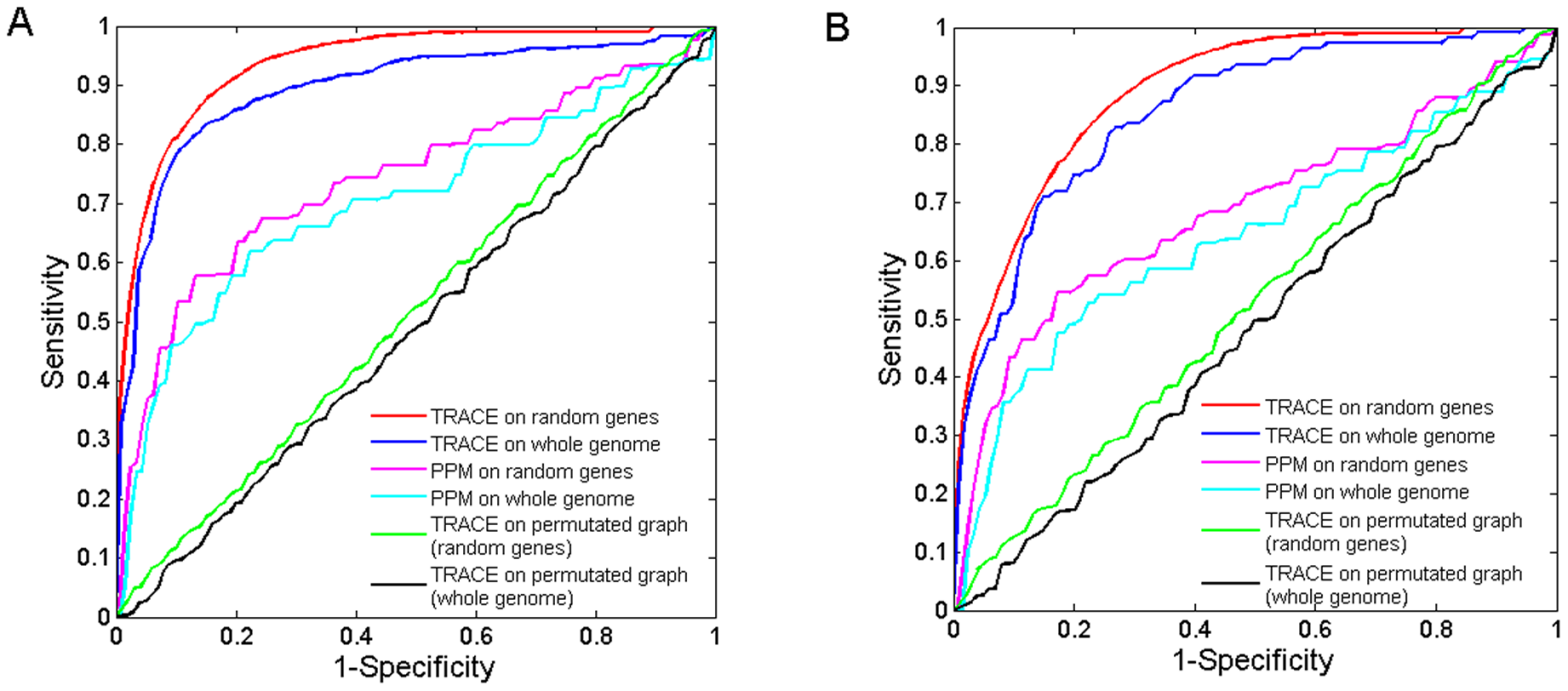
Predictive power of TRACE for enzyme function and components of pathways. (A) Validation of predicting enzyme function. (B) Validation results of predicting components of pathway. For both figures, ROC curves of TRACE were obtained for both random genes set and whole genome set. The results were compared between TRACE and PPM. In addition, the predictive power of TRACE was validated on a permutated network using both control sets.

To assess the performance of TRACE, two types of functionally related gene sets were used in the validation processes (see Methods and Materials for details). First, we used TRACE to identify enzymes with identical function. For the 2,135 RHA1 enzymes, 1,313 were classified into 338 EC categories, with each group containing at least 2 genes. When predicted enzyme functions on our random control gene sets, TRACE could achieve a TOP precision of 36% and AUC value of 93.5%, in which TOP is defined as the proportion of highest-ranked positive control genes, and AUC defined as the value of area under a receiver operating characteristic curve (see Methods and Materials for further explanation). Enzymes were assigned with an EC number according to experimental characterization, rather than sequence similarity alone [27], [28]. Therefore, the high precision achieved by TRACE indicated that it can predict gene function not solely based on sequence similarity. When TRACE was used to predict genes as part of the same pathway, it achieved a TOP precision of 24.5%, and an AUC value of 88.7%. Subsequently, the performance of TRACE was examined on the whole genome. For the prediction of enzyme functions as well as components of pathways, TRACE achieved a TOP precision as high as 32.7% and 21%, and an AUC value of 89.2% and 85.3%, respectively (Figure 1A,B). These values were only slightly lower than that of the random control, confirming that TRACE could obtain a relatively high level of precision for genome-wide scans as well.

To test if TRACE can utilize the topological information of the gene functional network, validations were executed on a permutated version of this network. The gene functional network was randomly permutated while keeping the same node degree distribution. The AUC values on two control gene sets for predicting enzyme functions and components of pathways were both decreased by almost 50% (Figure 1A,B). The performed benchmark tests suggested that our gene functional network stored important information on gene evolution, and that TRACE can effectively mine it to predict gene functional linkages.

### Comparison between TRACE and PPM

Whilst several methods for inferring gene functional linkages had been developed previously, few reported details related to the precision of prediction, mainly due to the lack of proper control sets and appropriate experimental tests [3], [6], [29]. To analyze the predictive power of TRACE over previously published tools, we compared TRACE with PPM [19] using our constructed control genes sets. For each gene, its phylogenetic profile was constructed as a 341 dimension 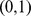 vector, where the value 0 indicates its absence and value 1 its presence in each genome. The correlation between any paired vectors was then calculated to describe their relationship. When predicting enzyme function on random control gene sets, PPM achieved a TOP value of 5.1% and an AUC value of 74%. On whole genome predictions, these values decreased to 3.2% (TOP) and 70.1% (AUC). When predicting components of pathways, the values for random control gene sets were 4.2% (TOP) and 68.6% (AUC). They decreased to 2.7% (TOP) and 64.3% (AUC) on whole genome predictions (Figure 1A,B). In conclusion, PPM results where lower compared to those yielded by TRACE, therefore TRACE's ability to predict gene functional linkages turned out to be superior.

### Computational prediction of a chromosome partitioning protein

**Figure 2:**
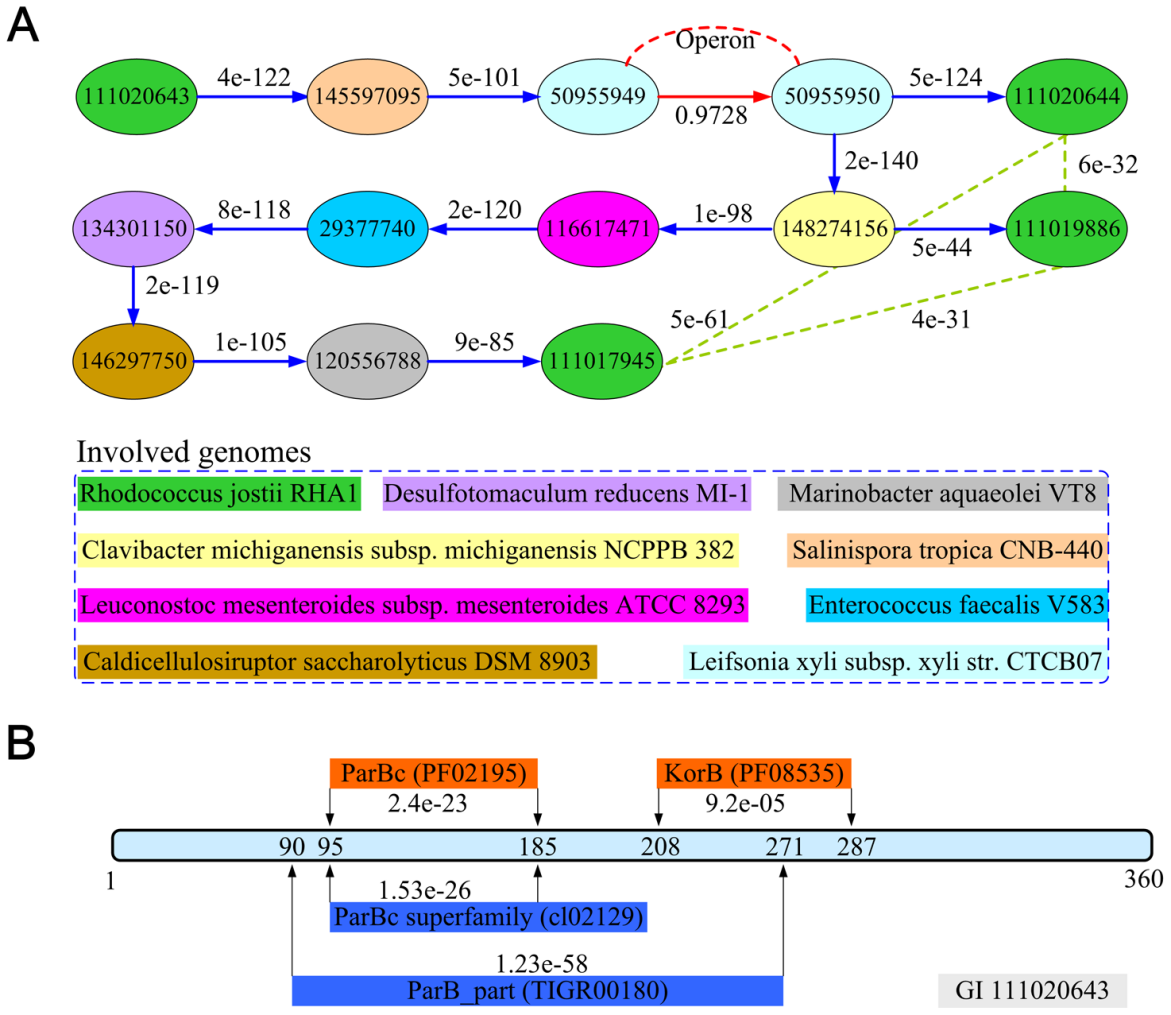
Computational analysis and domain prediction for R-ParB protein. (A) The top three ranked genes of *r-parB* and their connection details. In the connected paths, two operon genes 50955949 and 50955950 served as a key bridge link (red arrow). The identified three genes (111020644, 111019886, 111017945) exhibit sequence similarity between each other (green dotted line), but not with *r-parB*. The probability between genes in the same operon and sequence similarity (e-values retrieved from BLASTP) are presented. The genes and related genomes in connected paths are depicted in identical colours. (B) The predicted protein domains for gene 111020643 using the NCBI CDD and PFAM databases. There are two overlapping domains (blue) predicted by NCBI CDD database and two spatial separated domains (red) by PFAM database. The e-values were calculated by both databases.

To demonstrate the ability of TRACE to predict novel gene function linkages, we present a case study of gene ro03654 (GI 111020643) of RHA1, whose function was not well defined prior to our study. A genome-wide ranking of all other genes was performed. Amongst the 20 most highly-ranked genes, 7 genes were enriched in chromosome partitioning achieving a significant p-value of 3.2e-12 by the DAVID database [30] (Table S1). The top three-ranked genes were annotated as chromosome partitioning protein ParA (GI 111020644), ParA family chromosome partitioning ATPase (GI 111019886), and ParA family ATPase (GI 111017945). The connected shortest paths from ro03654 to these three genes were linked through an operon in *Leifsonia xyli subsp. xyli str.* CTCB07, suggesting that ro03654 was functionally related to the process of chromosome partitioning with high probability (Figure 2A). The sequences of these three genes were similar to each other, but had no similarity with ro03654. This result indicates that our TRACE algorithm can predict gene function by utilizing genomic context, in addition to sequence information. We further investigated the presence of protein domains within the ro03654 gene using both the NCBI CDD database [31] and the PFAM database [32]. From the NCBI CDD database, a ParB_part domain (TIGR00180) ranging from 90 to 271 bp was detected with an e-value of 1.23e-58 (Figure 2B). Within this region, a ParBc superfamily (cl02129) was further described with e-value of 1.53e-26. These predictions were confirmed by the PFAM database, and both domains were detected as ParBc (pf02195) from 95 to 185 bp and KorB (PF08535) from 208 to 287 bp. The ParB_part domain is a key functional domain described in many other ParB-like proteins [33], [34]. Thus, our results presented here clearly suggest that ro03654 is a chromosome partitioning-like protein. We therefore renamed it to R-ParB protein, in accordance with the nomenclature commonly agreed upon for this family of proteins.

### Verification of R-ParB biological function

**Figure 3:**
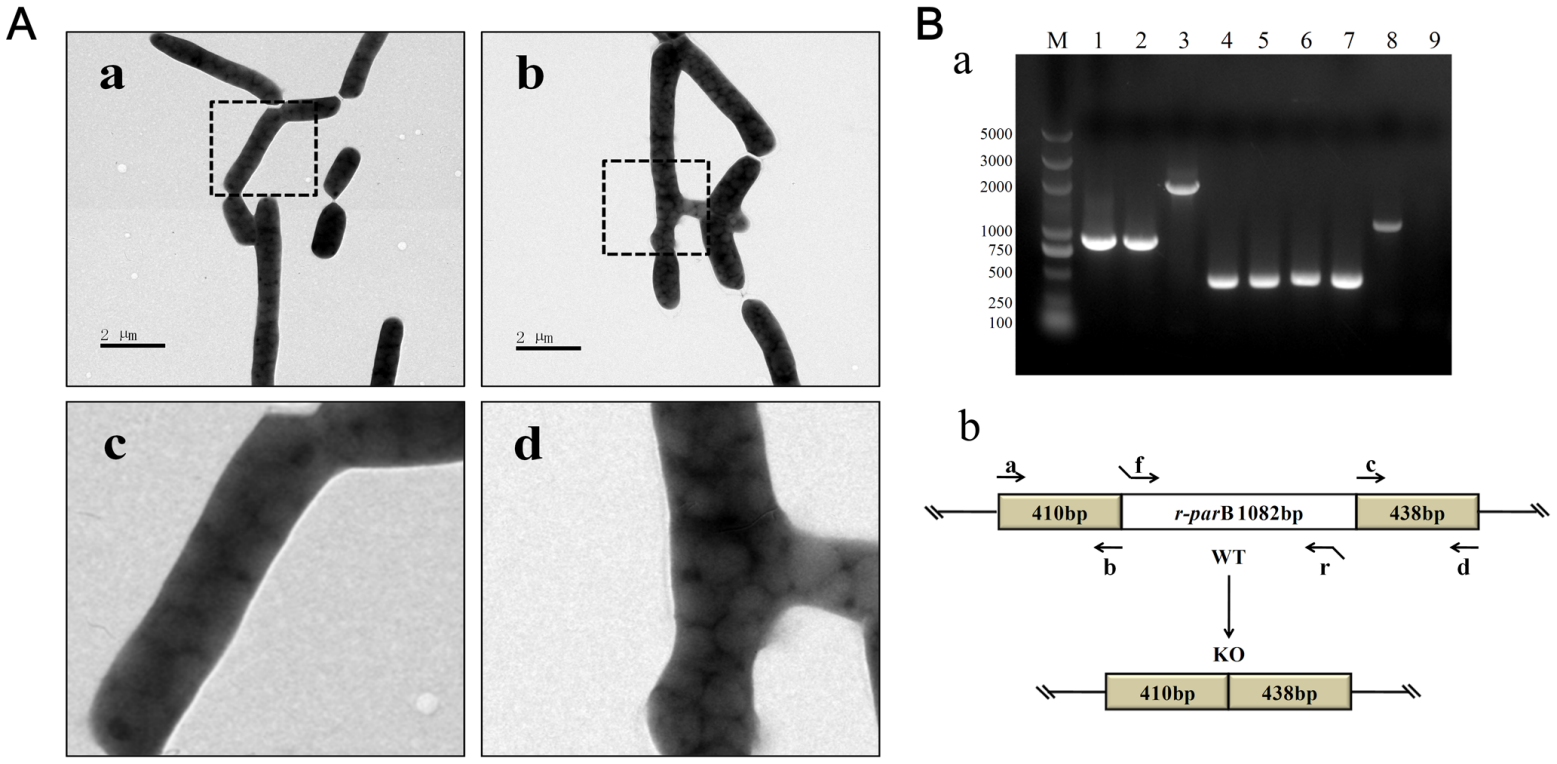
Morphology of the *parB* deletion mutant strain. (A) Transmission electron microscopy on RHA1 WT cells and *r-parB* deletion mutant strain. a, negative staining of RHA1-WT; b, negative staining of *r-parB* deletion mutants; c and d were the enlarged images of a and b, respectively. Bar  = 2 µm. (B) Identification of *r-parB* deletion mutant. a, PCR result of *r-parB* gene deletion. M, marker; lane 1, positive control of *r-parB* gene PCR fragment using primers r-parB-a/r-parB-d with knockout plasmid pK18mobsacB as template; lane 2, the PCR fragment of *r-parB* gene in the deletion mutant was 848 bp, and identical to the positive control; lane 3, the size of the WT fragment was 1930 bp; lane 4–5, the left flank sequences of AB were 410 bp used primers r-parB-a/r-parB-b with WT and deletion mutant cells as templates, respectively; lane 6–7, the right flank sequences of primers r-parB-c/r-parB-d, with templates as in lane 4–5; lane 8–9, r-parB-f and r-parB-r primers in *r-parB* gene sequence. b, diagram of *r-parB* gene deletion. Primers used as shown.

To confirm the function of R-ParB, genomic and biochemical experiments were performed in both wild type (WT) and a *r-parB* deletion mutant of RHA1. *Rhodococcus sp*. RHA1 is an important strain in the field of biofuel development, because it can synthesize and accumulate large mount of triacylglycerols (TAGs) [25], [26], [35]. RHA1 cells contain an intracellular organelle called lipid droplet that is the storage site for triacylglycerol (TAG) [36] (Figure 3A-a,c). *r-parB* was deleted by employing pK18*mobsacB* through homologous recombination (Figure 3B). In the deletion mutant, the flanking fragments between primers r-parB-a, r-parB-b and primers r-parB-c, r-parB-d remained identical, while the fragments between primers r-parB-a and r-parB-d were smaller than that of WT. At the same time, the PCR results using primers r-parB-f and r-parB-r indicate that the *r-parB* gene could be amplified by using WT genome as the template, but could not be amplified by using the deletion mutant genome as the template (Figure 3B-a). These results proved that a precise *r-parB* gene deletion mutant was generated (Figure 3B-b).

**Figure 4:**
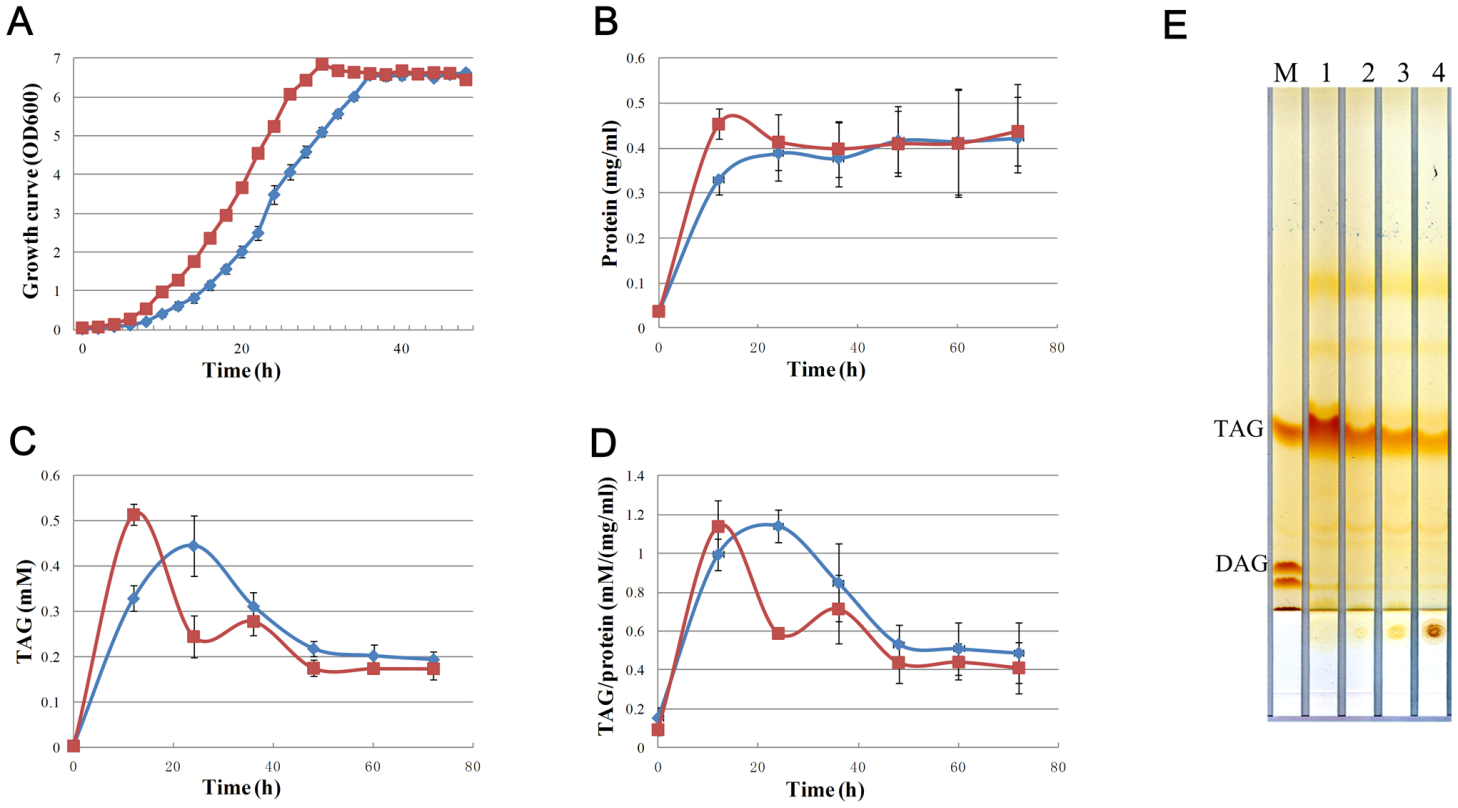
Growth rate comparison of RHA1 WT and a *r-parB* deletion mutant strain. (A) Growth curves for wild type (red curve) and *r-parB* deletion mutant (blue curve) in LB medium. (B) Protein content for RHA1-WT and *r-parB* deletion mutant. Bacterial samples were collected at different time points and washed twice with PBS before being dissolved in 1% Triton X-100 followed by sonication. Quantification of protein and TAG content was performed as described in Material and Methods. (C) TAG content of RHA1-WT and *r-parB* deletion mutant strains; identical amounts of cells were transferred into MSM medium after pre-culturing in LB. (D) TAG/protein content ratio for the two strains. (E) TAG was extracted from the same cell number, with cells cultivated in MSM for 24 h prior to TLC analysis. M, marker; lane 1, *r-parB* deletion mutant; lane 2, *r-parB* deletion mutant with pJAM2*-r-parB-egfp* over-expression plasmid; lane 3, RHA1-WT; lane 4, RHA1-WT with pJAM2*-parB-egfp* over-expression plasmid.

Phenotypically, the *r-parB* deletion mutant did not appear too different from WT, especially in terms of lipid droplet size and number as measured by EM imaging (Figure 3A-b,d). However, the growth rate of mutant cells differed substantially from that of RHA1 WT by comparing the protein and TAG content of RHA1 WT and *r-parB* deletion mutant respectively. To this end, cells of both strains were cultivated in MSM medium [37]. The growth curves indicated a clear delay of *r-parB* deletion mutant compared to RHA1 WT (Figure 4A). Protein content shows an obvious trend line, suggesting that the *r-parB* deletion mutant grew slower than RHA1 WT, especially at around 12 h (Figure 4B). Meanwhile, the TAG content exhibited a lower value at 12 h, though a higher value at the 24 h time point, and the peak value was smaller for the *r-parB* deletion mutant strain (Figure 4C). A similar phenomenon was observed for the TAG/protein content (Figure 4D). The delay of the peaks of TAG and TAG/protein content revealed the fact that in our experiments, cell division slowed down in the absence of the R-ParB protein.

pJAM2*-r-parB-egfp* fusion plasmid was constructed and over-expressed in *r-parB* deletion mutant and RHA1 WT to further test the TAG content dynamics and R-ParB protein location. *r-parB* deletion mutant and RHA1 WT were cultivated in LB for 24 h, and then transferred into MSM medium (1∶10) for another 24 h. All TAG contents were analyzed and compared with each other by TLC. The *r-parB* deletion mutant strain contained much more TAG than RHA1 WT at 24 h (Figure 4E) which was consistent with the results shown in Figure 4D. *r-parB* deletion mutant with over-expression of R-ParB-GFP fusion proteins had almost the same TAG content as RHA1 WT. RHA1 WT over-expressing R-ParB-GFP exhibited less TAG when compared with RHA1 WT itself. These results show that the TAG content within the *r-parB* deletion mutant could be restored by over-expressing R-ParB, thus providing confirmation of the function of R-ParB protein. Fluorescence microscope indicated that the R-ParB-GFP fusion protein was evenly distributed throughout the cytosol in both *r-parB* deletion mutant strain and RHA1 WT, similar to the GFP negative control (Figure S1).

Taken together, both the computational predictions and biological experiments strongly indicate that ro03654 is a ParB-like protein that is involved in chromosome partitioning. Its mutation caused a severe delay in cell division and a moderate delay of TAG peak from the 12 h time point (WT) to the 24 h time point. After 12 h, however, the TAG content was higher in the *r-parB* mutant when compared to the RHA1 WT, exhibiting a slow decrease compared to the two peaks that existed in RHA1 WT. The above results indicate that *r-parB* plays not only an important part in bacterial segregation, but also has an effect on TAG synthesis and accumulation. Our biological experiments thus confirmed the computational predictions performed using TRACE.

### Computational prediction and cell location of prokaryotic SNARE-like proteins

**Figure 5:**
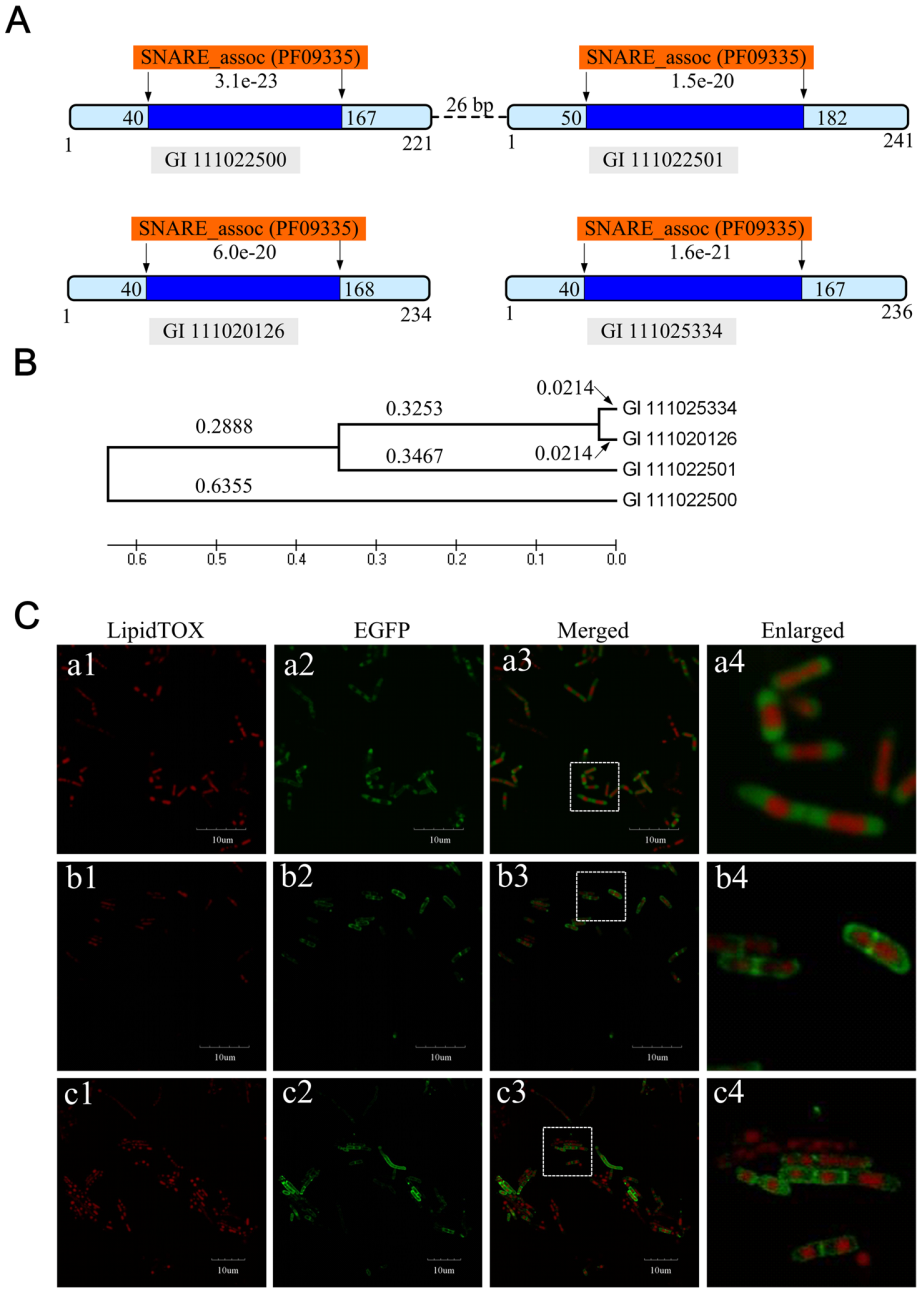
Plasma membrane location of SNARE-like proteins ro03137 and ro05535 in RHA1 cells. (A) Predicted domains of gene 111022500, 111022501, 111020126 and 111025334. Gene 111022500 and 111022501 were fused into one operon construct. They both include SNARE_assoc domains (PF09335) predicted by the PFAM database. (B) The minimum evolution tree shown for genes 111022500, 111022501, 111020126 and 111025334. The evolution distance (in billion years) and tree was calculated and constructed by using the maximum parsimony (MP) method embedded in MEGA4.0 [54]. (C) SNARE-like protein-coding genes ro03137 and ro05535 were amplified and inserted into the over-expression vector pJAM2*-egfp* to generate pJAM2-*ro03137-egfp* and pJAM2-*ro05535-egfp*. The plasmids were transformed into RHA1 WT cells by electroporation. Single colonies were picked, cultivated in LB for 48 h and transferred for cultivation into MSM for 24 h. Lipid droplets were stained by LipidTOX as described previously [36]. Cells were prepared for confocal microscopy as described in Materials and Methods. (a1–a4), over-expression of empty vector pJAM2-*egfp*; (b1–b4), over-expression of pJAM2-*ro03137-egfp*; (c1–c4), over-expression of pJAM2-*ro05535-egfp* (Bar  = 10 µm).

We showed that TRACE can also predict the functional modules that several proteins work together. To test this ability, the genes that can recall each other in top positions were analyzed. Interestingly, 4 hypothetical proteins ro05534 (GI 111022500), ro05535 (GI 111022501), ro03137 (GI 111020126) and ro08552 (GI 111025334) were identified as SNARE-like proteins. In eukaryotic cells, SNARE-like proteins play an important role in membrane and lipid droplet fusion [38], [39]. In our computational analysis, these four proteins recalled each other in top 3 positions, suggesting they are functionally related. All four proteins were identified as containing SNARE_assoc domains. Gene ro05534 and ro05535 were grouped into an operon structure (Figure 5A). Phylogenetic analysis resulted in classification of these four proteins into two evolutionary groups. Genes 111022500 and 111022501 were estimated to have diverged approx. 0.6355 billion years ago. Genes 111022500, 111025334 and 111020126 were clustered into one group, whilst genes 111025334 and 111020126 were duplicated more recently (Figure 5B). Earlier studies revealed that eukaryotic SNARE proteins are assembled into protein complexes, consisting of v-SNARE and t-SNARE [40], [41]. Our prediction and analysis of 4 SNARE-like proteins of RHA1 presents a possible starting point for the evolution of SNARE protein complexes from prokaryotes to eukaryotes.

We tested the localization of the SNARE-like protein ro05535 and ro03137 by generating over-expression plasmids pJAM2*-ro05535-egfp*, pJAM2*-ro03137-egfp*. Positive clones of RHA1 expressing the fusion proteins ro05535-EGFP and ro03137-EGFP were analyzed by confocal microscope. Interestingly, these two SNARE-like proteins were evenly distributed on the plasma membrane, a phenomenon that was not observed for EGFP vectors alone (Figure 5C). The location at the plasma membrane was consistent with earlier observations made in eukaryotic cells [38], [39], [42]. In conclusion, our data suggest that the prokaryotic SNARE-like proteins might play an important role in plasma membrane activity.

## Discussion

A computational method TRACE was developed to infer novel gene functional linkages at the level of prokaryotic genomes. Validation experiments confirmed that TRACE is able to achieve high levels of prediction accuracy. The main advantage of this newly developed analysis tool results from its effective tracing ability of gene-related evolutionary information using a constructed gene functional network. Whereas an earlier proposed PPM algorithm only achieves approximate clues for gene functional relationships, TRACE obtained optimal solutions applying an exact graph search method. Serving as an unsupervised method, TRACE voided the heavy burden of training data selection and parameter optimization.

Gene ro03654 (GI 111020643) of RHA1 was successfully predicted as representing a *parB*-like protein. Biochemical and molecular experiments were performed to test if its function is related to chromosome partitioning. The faithful replication and proper segregation of chromosomes and plasmids are essential for the normal growth and development of eu- and prokaryotic cells, and ParB proteins cooperate with various proteins such as ParA, ParM, ParR, TubZ and TubR, all important for moving newly replicated chromosomes or plasmids to the opposite sides of the division plane in bacterial cells [43]–[48]. Our experiments confirmed that deletion of *r-parB* in RHA1 causes severe disruption of cell division, and also effects TAG accumulation. Because RHA1 is one of most important oleaginous strains for TAG production, these results may present a novel way in controlling neural lipid synthesis, accumulation and degradation.

In addition, ro05534, ro05535, ro03137 and ro08552 were predicted to be SNARE-like proteins involved in membrane fusion processes in mammalian systems. Two proteins (ro05535 and ro03137) were observed to be localized at the plasma membrane when over expressed, indicating that the SNAREs are functionally conserved in membrane dynamics. Our results of these SNARE-like proteins contribute to their functional study in prokaryotic cells, and will hopefully help us understand their evolution from prokaryotes to eukaryotes.

To further improve the precision of TRACE, integrating different types of information will be a feasible approach, as recently highlighted for the field of integrated systems biology [49]. Although the edges were weighted by sequence similarity and operon probability in our study, they can surely be weighted by other information, such as gene co-expression. Design more effective network searching algorithms to infer relationships among genes through the gene functional network are also desired. We are hopeful that future analysis of gene connecting paths outputted by TRACE will shed light on the evolutionary analysis of gene and biological processes.

## Materials and Methods

### TRACE design

**Figure 6:**
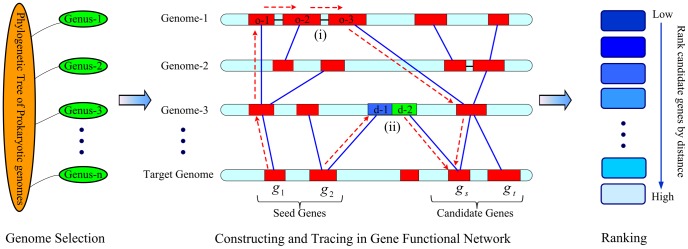
Schematic view of the TRACE method. Prokaryotic genomes were selected from each genus in order to ensure evolutionary distances. A gene functional network was constructed by defining the similarity weight and operon weight. The shortest path values were then calculated from seed genes to each candidate gene. (i) shows functional connections passed on by three genes o-1, o-2, o-3 in an operon. (ii) shows functional connections passed on by two domains, d-1 and d-2. Whole genome genes were ranked by shortest path distances and the highest-ranked gene was considered to be its functional linkage gene.

Our design of TRACE was based on the assumption that the evolutionary information of functional related genes is stored in different genomes. It was programmed to perform three essential steps to predict gene functional linkages by tracing evolutionary information. First, 341 genomes were selected to construct a gene functional network, where each gene was taken as a node and edges were weighted by transforming gene similarity and operon probability. To avoid redundant information, strains were selected following a critical rule that each genome with the longest sequence was selected from each genus. Genus was defined as a relative evolutionary distance in the bacterial kingdom. The longest genome will be benefit to the abundance of genes, because a longer genome usually includes more genes. Second, the shortest path value between a gene pair was calculated as their functional distance. This process can effectively utilize the evolutionary events (separation and fusion of operons and domains) of different genomes. Third, given a gene, all other genes in the same genome were ranked according to their functional distances. The most highly ranked genes were considered to be in the same gene functional linkage (Figure 6). The details for network construction and calculations are described below.

### Construction of the gene functional network

341 genomes (658 genomes and plasmids, Table S2) of different genera were selected and downloaded from the NCBI database (NCBI release of July 2012). All the genes' GI IDs used in the paper are NCBI protein IDs. In total 1,069,928 genes of 341 genomes were compared with each other using BLASTP [50]. All operons within each genome were predicted by our operon prediction program as described earlier [14] and can be downloaded from the operon prediction database (DOOR, http://csbl1.bmb.uga.edu/OperonDB_10142009/DOOR.php) [15].

Subsequently, 1,069,928 genes were used to construct a gene functional network, within each gene was defined as a node. The edge between two nodes was weighted by transforming the sequence similarity or operon probability as follows:

For each pair of genes 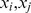, sequence similarity weight is defined as:
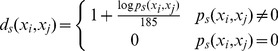
(1)where 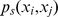 is the BLAST e-value for genes 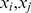. Since the e-values less than 1e-185 are set as 0 in the BLAST program [50], the number 185 served as a normalization factor. It is clear that 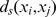 has a value between 0 and 1. The more similar two genes are, the smaller the 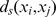 value is.

The operon weight 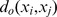 is defined as follows:
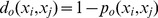
(2)Where 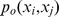 presents the probability of 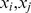 being part of the same operon. This was calculated by our operon prediction program [14], which is considered one of most reliable operon prediction methods [51]. 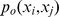 ranges from 0 to 1, and the higher the value is, the higher the probability that a pair of genes are part of the same operon. The operon weight is valued between 0 to 1. The higher the probability of two genes to be in the same operon, the smaller the value 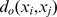.

### Calculating the functional distance of a gene pair

After we constructed a gene functional network, the shortest path method [52] was used to calculate the functional distance between genes of the same pair. Given a seed gene 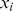 and a candidate gene 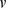, the functional distance is defined as the shortest path value between them: 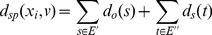
(3)where 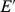 denotes edges with operon weight and 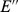 for those with similarity weight. If there is more than one seed gene, the values of seed genes to candidate gene are summed and averaged. 
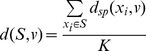
(4)where 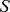 is the seed gene set and 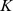 is its item number.

### Computational cross validation of TRACE

Two types of functionally related gene sets were used for large-scale validation. First, genes with the same EC number were defined as a functional related gene set. For the RHA1 genome, 2,135 genes were assigned 831 EC numbers. 338 EC groups consisted of more than 2 genes, and added up to 1,313 genes in total. Second, genes involved in the same pathway were defined as a functionally related gene set. Using the KEGG database, 5,901 genes were identified as members of 112 pathways (including more than 2 genes) for RHA1. All enzyme EC annotations and pathway information were obtained from the KEGG database (released July 2011) [53].

To examine the capability of TRACE in discovering genes with previously identified relationship, two types of ‘leave-one-out’ cross-validation experiments were conducted. Given a functionally related gene set, one gene was selected to serve as positive control and the remaining genes were used as seed genes. The positive control gene was then grouped with randomly selected 99 genes as random control genes, or alternatively grouped with all other genes of the RHA1 genome to act as whole genome control genes. The positive control gene was assumed functional unknown by deleting all its edges to seed genes in our gene functional network. In each validation, a positive control gene was prioritized against random control genes or whole genome control genes. In each computational run, if the positive control gene was ranked as highest, it was considered a successful prediction.

Two criteria were used to evaluate the performance of TRACE. Taking the cross-validation against random control genes as an example, we were able to obtain a ranking list after each validation run. The proportion of highest-ranked positive control genes was calculated as Top One Precision (TOP). Second, a Receiver Operating Characteristic Curve (ROC), a graphical plot used to illustrate the performance of a binary classifier system upon changing of the discrimination threshold, was drawn and the area under the curve (AUC) was calculated. Larger TOP and AUC values thus indicate higher performance of the prioritization method.

### Strains and culture conditions of RHA1

The strains and plasmids used in this study are listed in Table 1. RHA1 cells were cultivated aerobically for 48 h in Luria-Bertani (LB) in Erlenmeyer flasks at 30°C. To promote accumulation of triacylglycerols (TAG), 10 ml cells (OD600≈1.5) were harvested by centrifugation and then cultivated for 24 h in 100 ml mineral salt medium (MSM) with 0.5 g/l NH_4_Cl as a nitrogen source and 10 g/l gluconate sodium as carbon source.

**Table 1:** Strains and plasmids used in this study.

Strains and Plasmids	Characteristics	Sources or References
*Rhodococcus* sp. RHA1	Wild type	Lindsay Eltis, University of British Columbia, Canada
*Rhodococcus* sp. RHA1-*Δr-parB*	RHA1 with *r-parB* deletion	This study
DH5α	Host used for plasmid cloning	This laboratory
pK18*mobsacB*	5.7-kb mobilizable suicide vector used for genome deletion	Ping Xu, Shanghai Jiao Tong University, China
p*Δr-parB*	849 bp fusion PCR fragment flanking *r-parB* cloned into pK18mobsacB used to make *r-parB*	This study
pJAM2-*egfp*	A shuttle vector between E.coli and Rhodococcus which can express carried genes	Alexander Steinbüchel, University of Münster, Germany
pJAM2-*r-parB-egfp*	Expresses R-ParB-EGFP fusion protein	This study
pJAM2-*ro03137-egfp*	Expresses ro03137-EGFP fusion protein	This study
pJAM2-*ro05535-egfp*	Expresses ro05535-EGFP fusion protein	This study

### Construction of the *r-parB* deletion mutant

The plasmid used for mutagenesis was constructed as follows. The upstream sequence of *r-parB* was amplified using primers r-parB-a and r-parB-b with the *Eco*R I site at the 5′ terminus to generate a fragment AB. Similarly, the downstream sequence was amplified using primers r-parB-c and r-parB-d with the *Hin*d III site at the 3′ terminus to generate fragment CD. Fragments AB and CD was annealed at their overlapping region and amplified by primers r-parB-a and r-parB-d as a single fragment KO-AD. Primers used are listed in Table 2. After sequencing, the deletion fragment KO-AD was cloned into the pK18*mobsacB* plasmid and transformed into RHA1 cells using a Bio-Rad 165–2100 MicroPulser (Bio-Rad, USA). The clones were selected on LB agar plates containing 30 µg/ml nalidixic acid and 50 µg/ml kanamycin followed by *sacB* counter-selection on 10% sucrose plate. Kanamycin-sensitive and sucrose-resistant clones were further confirmed by PCR using primers r-parB-a, r-parB-b, r-parB-c, r-parB-d, r-parB-f, and r-parB-r.

**Table 2:** Primers used in this study.

Primer Name	Primer Sequence
r-parB-a	5′-CGGAATTCGAAGGCTGGTGAGGAGGACAGCGT-3′
r-parB-b	5′-CCATCCACTAAACTTAAACTGACGGACCGACTCCAGCAGAACTA-3′
r-parB-c	5′-GTTTAAGTTTAGTGGATGGTGACTCATCGCTGCTCCTGCTTCGTG-3′
r-parB-d	5′-CACAAGCTTCCGACTTCATCCTGATCGACTGCCC-3′
r-parB-f	5′-GCGGATCCAGTCAGACGCGTAAGGGTGGACTTG-3′
r-parB-r	5′-GCGGATCCTGAATCGGCCTTCTGTGCCTCCAT-3′
ro03137-F	5′-ATAGGATCCAATGTGGCACTGGGACTGGGCTTAC-3′
ro03137-R	5′-ATAGGATCCTCGGGTGAATGGTGCGCCGG-3′
ro05535-F	5′-CGGGATCCCAGGCAGCATCGGATTCCGTGA-3′
ro05535-R	5′-CGGGATCCGGACGTCGGATCGGTGGGGGTG-3′

### Construction of GFP fusion proteins

*r-parB*, ro03137, ro05535 were amplified using the primers shown in Table 2 without their native start- and stop-codons. The fragments were then cloned into the *Bam*HI site of the vector pJAM2-*egfp*, generating over-expression plasmids pJAM2- *r-parB*-*egfp*, pJAM2-*ro03137*-*egfp* and pJAM2-*ro05535*-*egfp*. The plasmids were transformed into RHA1 using a Bio-Rad 165–2100 MicroPulser (Bio-Rad, USA).

### Confocal microscopy and electron microscopy

RHA1 cells were washed twice with PBS and then mounted onto coverslips pre-treated with rat tail collagen. Samples were dried for 30 min at room temperature prior washing with 1 ml PBS. Samples were then incubated for 30 min in a 1∶500 solution of LipidTOX Red under darkness at room temperature, and subsequently mounted onto glass slides with Mowiol mounting media and analyzed by confocal microscopy (Olympus FV1000).

Negative staining was used to obtain bacterial images. First, cells were loaded onto carbon-coated copper grids, and then stained for 2 min using 2% (w/v) phosphotungstic acid. Finally, the grids were washed three times with deionized water. Images were taken using a FEI Tecnai 20 electron microscope (FEI Co., Netherlands).

### TAG measurements

Equal volumes of RHA1 WT cells and *r-parB* (GI 111020643) deletion mutant grown in LB were transferred into MSM (1∶10 v/v). A 1 ml cell suspension was taken at different time points. After washing twice with 1 ml PBS, cells were dissolved in 200–400 µl 1% Triton X-100 and probe-sonicated for 6 times 6 second at 200 Watt (Cole-Parmer, USA). TAG concentration was measured by using triglyceride assay kit E1003 (Applygen Technologies, China). Proteins content was quantified by using Pierce BCA protein Assay Kit (Thermo, USA).

### Thin layer chromatography (TLC)

Cells were cultivated in LB medium for 48 h and transferred into MSM medium (1∶10, v/v). The neutral lipids of the cells were extracted twice by chloroform:acetone:medium (1∶1∶1, v/v/v). The organic phases were then collected and dried under high purity nitrogen gas. The total lipids were dissolved in 100 µl chloroform, vortexed and centrifuged for 1 min at 10,000 g. Whatman Purasil™ 60 FÅ silica gel plates (Merck, Germany) were utilized to perform TLC analysis. The extracted lipids were separated using a solvent system of hexane: diethyl ether: acetic acid (80∶20∶1, v/v/v). The plates were visualized by iodine vapor following complete drying.

## Supporting Information

Figure S1 **Cytosolic location of R-ParB-GFP.** All cells were cultivated in MSM for 24 h, and then washed twice by PBS. Lipid droplets were stained by LipidTOX as described previously [36]. Images were taken by confocal microscopy. a1–a4, RHA1-WT overexpressed with the vector pJAM2-egfp; b1–b2, RHA1-WT overexpressed with the vector pJAM2-r-parB-egfp; c1–c4, r-parB deletion mutant overexpressed with the vector pJAM2-egfp; d1–d4, r-parB deletion mutant overexpressed with pJAM2-r-parB-egfp. Bar  = 10 µm.
(TIF)

Table S1 **Detailed information for the TRACE analysis of top 20 ranked genes of R-ParB protein (GI 111020643).**
(XLS)

Table S2 **List of 341 genomes used in constructing the gene functional network.**
(XLS)
