# Supplementary figures and images for: Tracing Evolutionary Footprints to Identify Novel Gene Functional Linkages
Source: PLoS One. 2013 Jun 25;8(6):e66817. doi: 10.1371/journal.pone.0066817 (PMC3692504; doi:10.1371/journal.pone.0066817)

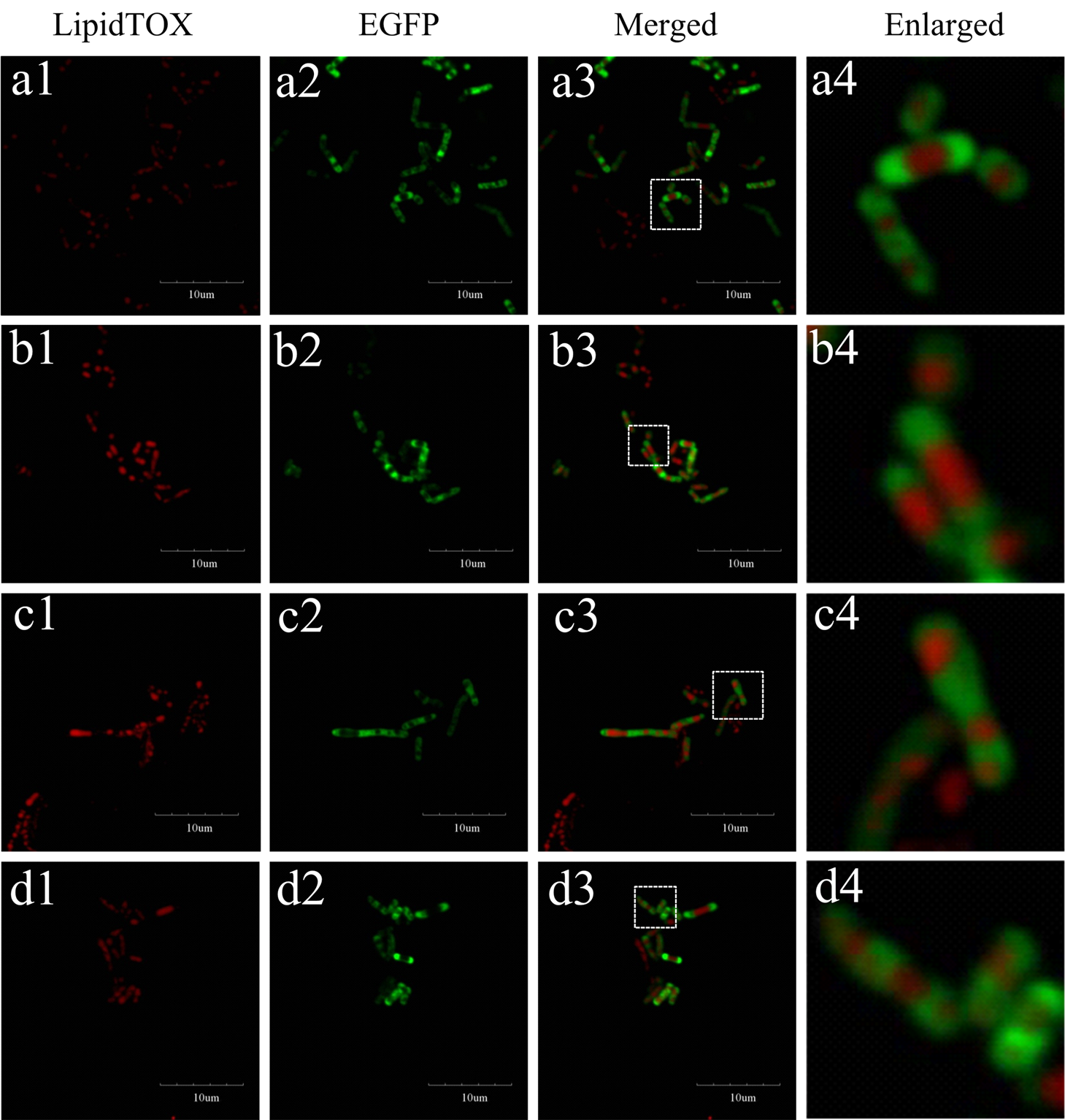

Supplement: Figure S1 — Cytosolic location of R-ParB-GFP. All cells were cultivated in MSM for 24 h, and then washed twice by PBS. Lipid droplets were stained by LipidTOX as described previously [36]. Images were taken by confocal microscopy. a1–a4, RHA1-WT overexpressed with the vector pJAM2-egfp; b1–b2, RHA1-WT overexpressed with the vector pJAM2-r-parB-egfp; c1–c4, r-parB deletion mutant overexpressed with the vector pJAM2-egfp; d1–d4, r-parB deletion mutant overexpressed with pJAM2-r-parB-egfp. Bar = 10 µm. (TIF) [file pone.0066817.s001.tif]
